# Neurobehavioral and developmental profiles: genotype–phenotype correlations in individuals with Cornelia de Lange syndrome

**DOI:** 10.1186/s13023-024-03104-1

**Published:** 2024-03-10

**Authors:** Rowena Ng, Julia O’Connor, Deirdre Summa, Antonie D. Kline

**Affiliations:** 1 Department of Neuropsychology, Kennedy Krieger Institute, 1750 E. Fairmount Ave, Baltimore, MD 21231 USA; 2 Department of Psychiatry and Behavioral Sciences, Johns Hopkins University School of Medicine, Baltimore, MD USA; 3 Cornelia de Lange Syndrome Foundation, Avon, CT USA; 4 Harvey Institute for Human Genetics, Department of Pediatrics, Greater Baltimore Medical Center, Baltimore, MD USA

**Keywords:** Genetics/genetic disorders, NIPBL, SMC1A, Cornelia de Lange syndrome, Development, Behavior functioning, Interventions

## Abstract

**Background:**

Cornelia de Lange (CdLS) is a rare genetic disorder that affects most body systems. Variants in multiple genes including *NIPBL* and *SMC1A,* can cause the syndrome. To date, literature on genotype–phenotype associations in individuals with CdLS is extremely limited, although studies suggest some differences in clinical phenotype severity across variants. This study aimed to examine and compare neurobehavioral differences and developmental variability across CdLS genes, specifically *NIPBL* and *SMC1A,* and identify genotype–phenotype correlations.

**Participants and methods:**

This patient-reported outcomes study included accessing data from the Coordination of Rare Diseases registry at Sanford. Parents of a total of 26 children/adults with CdLS and a known variant in *NIPBL* (Mean age = 20.46 years, SD = 11.21) and 12 with a known variant in *SMC1A* (Mean age = 11.08 years, SD = 9.04) completed a series of questionnaires regarding their child’s developmental history. This included attainment of common language and motor milestones, intervention history, and behavior functioning. Developmental history and reported behavior regulation difficulties were compared across variant groups.

**Results:**

Overall, individuals with a pathogenic variant in *NIPBL* or *SMC1A* were similarly delayed across motor and language milestones with about 70% not using phrase speech and 30–50% not walking by 5 years of age. However, those with *NIPBL* variants showed more severity in behavioral phenotype, namely with more repetitive behaviors, tantrums, and withdrawn behaviors. In addition, these individuals were more likely than those with *SMC1A* variants to demonstrate self-injurious behaviors, and anxiety. Both groups yielded a similar proportion of participants who participated in speech and occupational therapy, however those with *SMC1A* variants were more likely to engage in physical therapy. Both clinical groups report low rate of communicative or assistive device use despite a large proportion of participants never mastering single word or sentence use.

**Conclusions:**

Study results are consistent with recent investigations highlighting more severe behavioral phenotype, particularly autistic features, anxiety, and behavior regulation challenges, among those with *NIPBL* variants albeit comparable developmental milestones. Both groups endorsed very elevated attention problems. Findings highlight importance of early interventions, including behavioral health services.

## Introduction

Cornelia de Lange syndrome (CdLS; MIM 122470) is a rare neurogenetic disorder caused by pathogenic variants in genes related to transcription regulation, particularly cohesin functions. The syndrome is typically characterized by limb abnormalities, growth and developmental delays, intellectual disability, unique dysmorphic facial features and multisystem impairment [15, 13]. Affected individuals with CdLS commonly have behavioral issues, presenting with autistic features, primarily repetitive or inflexible behaviors [4, 20], anxiety [17], attention problems, hyperactivity and self-injurious behaviors [4]. Notably, a recent consensus statement on the diagnosis and clinical management of CdLS has implicated both classic and atypical forms of CdLS, contending a spectrum of phenotypic features [14].

Molecular genetic investigations have revealed that multiple genetic variants can cause the classic or atypical CdLS phenotypes. A pathogenic variant in *NIPBL* is responsible for the majority of cases of CdLS, more commonly those with classic syndrome phenotype (~ 60–70%, [14]). Variants in *SMC1A* make up about 5% of those with CdLS [11], largely presenting with the non-classic form, although there is a group of individuals with loss of function variants in *SMC1A* presenting with a Rett syndrome-like phenotype [5]. Variants in *RAD21, SMC3, BRD4,* and *ANKRD11* have also been seen among those with atypical CdLS [3, 7, 9, 10, 18], while pathogenic *HDAC8* variants have been linked to both classic and non-classic forms [3]. In effect, classic CdLS is more commonly seen from variants of proteins that regulate cohesin functioning (*NIPBL, HDAC8*), whereas non-classic or milder CdLS phenotypes are often attributed to variants in genes that encode structural units of the cohesin complex (*SMC1A, RAD21, SMC3*) or cohesin-associated proteins (*ANKRD11, BRD4*, and others).

Investigations over the years have consistently reported variability in the severity of the clinical phenotype across gene variants causative of CdLS. Individuals with non-classic CdLS tend to present with less severe limb or structural abnormalities [2, 8, 10, 16, 19], craniofacial profile [2, 8, 10, 16], growth delays (e.g., weight, head circumference, [8, 10, 16]), and cognitive challenges [7, 9, 10]. However, to our knowledge, to date, the genotype–phenotype association in neurobehavioral and developmental profiles of CdLS remains a gap in literature. Providing more precise phenotype characterization across genotype enables more focused clinical care and can shed light onto the complex psychophysiological mechanisms underlying the range of CdLS presentations.

Accordingly, this study examines the behavior, developmental and intervention histories of 38 individuals with CdLS (26 with a pathogenic variant in *NIPBL,* 12 with an *SMC1A* variant). Data utilized in this study stem from developmental inventories completed by caregivers who participated in the Coordination of Rare Diseases (CoRDS) registry at Sanford. Consistent with the body of literature that highlights more severe clinical symptoms among those with *NIPBL* than those with other CdLS-causative variants [12, 14], we hypothesized that those with a pathogenic variant in *NIPBL* will present with more severe developmental delays, enroll in more intervention services (speech, occupational, and/or physical therapies) and demonstrate more challenging behaviors than those with an *SMC1A* variant.

## Methods

### Clinical sample and procedures

Data of 38 individuals with CdLS from the CoRDS registry were included in this study, 26 with a pathogenic variant in *NIPBL* and 12 with one in *SMC1A.* Information regarding the variant type was not collected by the registry. Participant characteristics and behavioral/attention composite scores are outlined in Table 1. Although both groups yielded similar range in age of participants, the *NIPBL* group was generally older (*NIPBL:* Mean age = 20.46 years, SD = 11.21, range 5–36; *SMC1A:* Mean age = 11.08 years, SD = 9.04, range = 5–38; F = 6.42, *p* = 0.016, η^2^ = 0.15). Racial composition between the two groups differed. The *SMC1A* group had more diverse representation whereas participants with *NIPBL* variant are all White. Both groups were comparable in other sociodemographic information including sex and insurance coverage, which was considered a proxy of socioeconomic status. All participants reside in the United States.

**Table 1:** Participant characteristics, intervention history, and behavioral functioning

	NIPBL (N = 26)	SMC1A (N = 12)	*p* Value
	Mean (standard deviation)		
Sex	10F	6F	n.s
Age (year)	20.46 (11.21)	11.08 (9.04)	F = 6.42, *p* = 0.016
Race			
White	100%	75%	FET *p* = 0.026
Asian	0%	16.7%	
Other	0%	8.3%	
Insurance			
Private/commercial	46.2%	66.7%	n.s
Medical assistance	34.6%	8.3%	
Military	11.5%	16.7%	
History of self injurious behaviors (% of the sample)	58.3%	0%	FET *p* = 0.002
History of anxiety (% of the sample)	70.8%	16.7%	FET *p* = 0.003
Attention total (minimum to maximum score = 0–8)	N = 20, 4.90 (2.38)	N = 10, 5.00 (2.53)	n.s
Repetitive behavior total (minimum to maximum score = 0–8)	N = 22, 2.77 (2.26)	N = 11, 0.81 (1.25)	U = 55.5, *p* = 0.01
Intervention history (% of sample, participation on a weekly to monthly basis)			
Speech/language therapy	50%	77.8%	n.s
Occupational therapy	59.1%	80%	n.s
Physical therapy	45.5%	90%	FET *p* = 0.04
Total therapies (minimum of 0, maximum of 3)	1.54 (1.40)	2.40 (1.07)	n.s
Uses communication devices	3.8%	25%	FET *p* = 0.08

*FET* Fishers exact test, *n.s.* not significant results (*p* > 0.10)

Only respondents who completed all attention and repetitive behavior items were included in the mean average for Attention Total and Repetitive Behavior Total

Patient-reported data from CoRDS (https://research.sanfordhealth.org/rare-disease-registry) were accessed as part of this study. Caregivers and parents of individuals with a diagnosis of CdLS age 5 years and older completed a battery of surveys to obtain natural history. Demographic, medical, neurologic, developmental and behavioral information were inquired across the inventories. Caregivers were asked to indicate the timeframe when their child achieved a milestone. The response options available were to check “< 12 months” or “other”, which subsequently prompted them to list the age (years) during which the child met the milestone. Participating caregivers also completed four rating items indexing attention and another four focused on repetitive behaviors, all of which were on a 3-point Likert scale (0 = Never a problem, 1 = Not a problem today but in the past, 2 = Currently a problem). The surveys that caregivers completed were designed by both clinical and scientific advisors from the Cornelia de Lange Foundation (CdLS) and CoRDS, a non-profit organization that works with patient advocacy groups and researchers to collect clinical information related to rare diseases in a standardized fashion.

Exclusion criteria primarily consisted of individuals who did not report genetic variant resulting in the CdLS diagnosis. This study protocol was approved by the Institutional Review Board at Johns Hopkins Medicine.

### Data strategy

The first author analyzed the anonymized patient data with SPSS 26.0. The number of patients who reported weekly to monthly participation in speech/language, occupational, and physical therapies was tabulated and summed into Total Intervention Use (e.g., 0 refers to no inventions, 3 refers to participation in all three therapies). Likewise, the proportion of patients who met a language or motor developmental milestone by the first 5 years of life was tallied. Ratings for the 8 items indexing attention and rigid behaviors were aggregated into two composite scores (Attention Total, Repetitive Behaviors Total). History of self-injurious behaviors and anxiety were dichotomously coded (yes/no significant history).

Non-parametric (Mann Whitney U test) was used to examine group differences in age at meeting developmental milestones, attention/behavior composites as well as cumulative intervention use given tests of normality suggested data were not normally distributed. Linear regression models were used to examine the association between genotype and language/motor developmental milestones after controlling for age at survey completion. Fishers exact test was applied to determine group differences in sociodemographic variables, the proportion of patients who never mastered a language/motor skill, and the proportion of patients endorsing specific attention, hyperactive and repetitive behaviors.

## Results

### Developmental milestones and intervention services

**Fig. 1:**
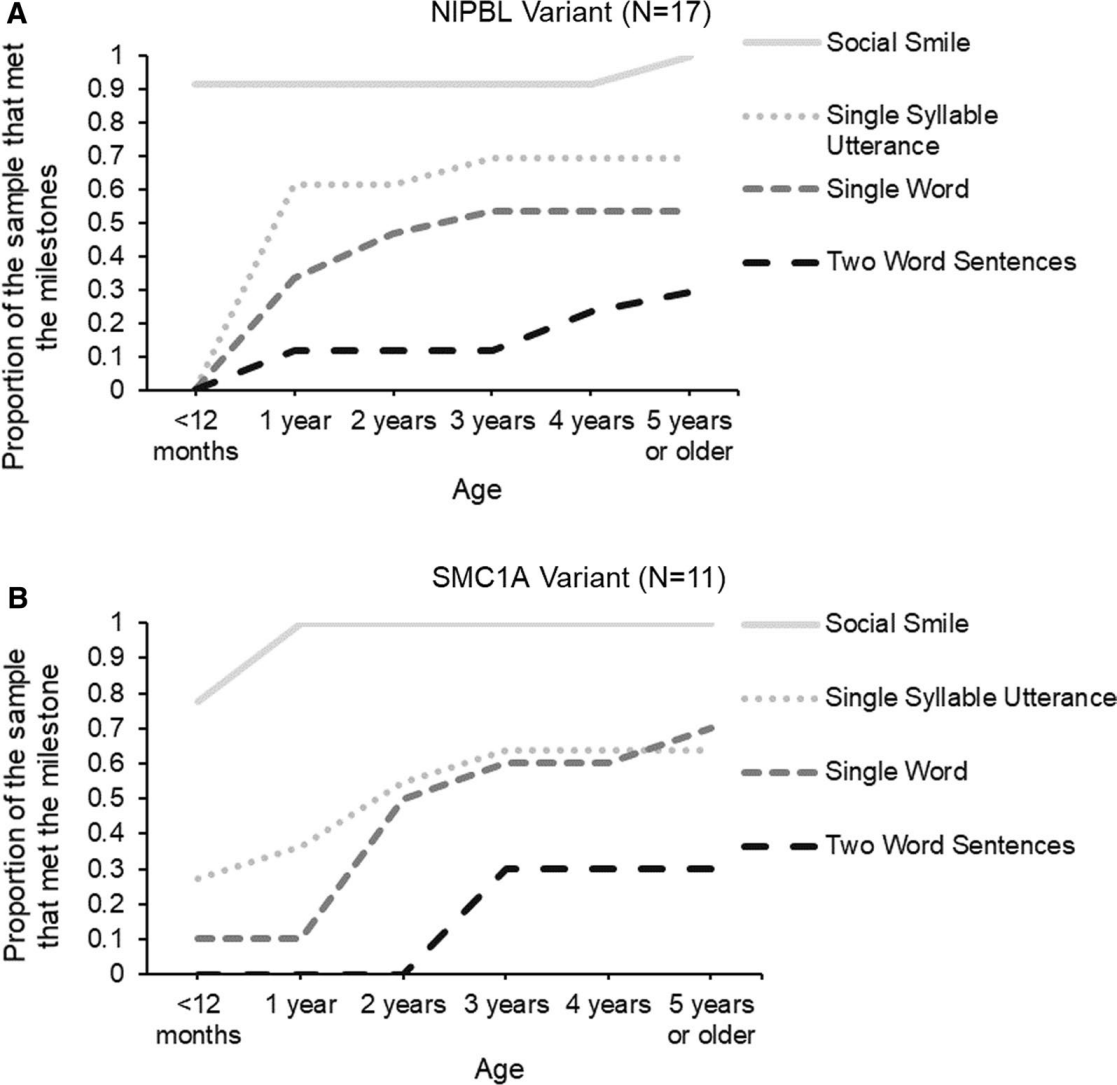
**A** and **B** Proportion of the patients who met the language developmental milestones by 5 years of age

**Fig. 2:**
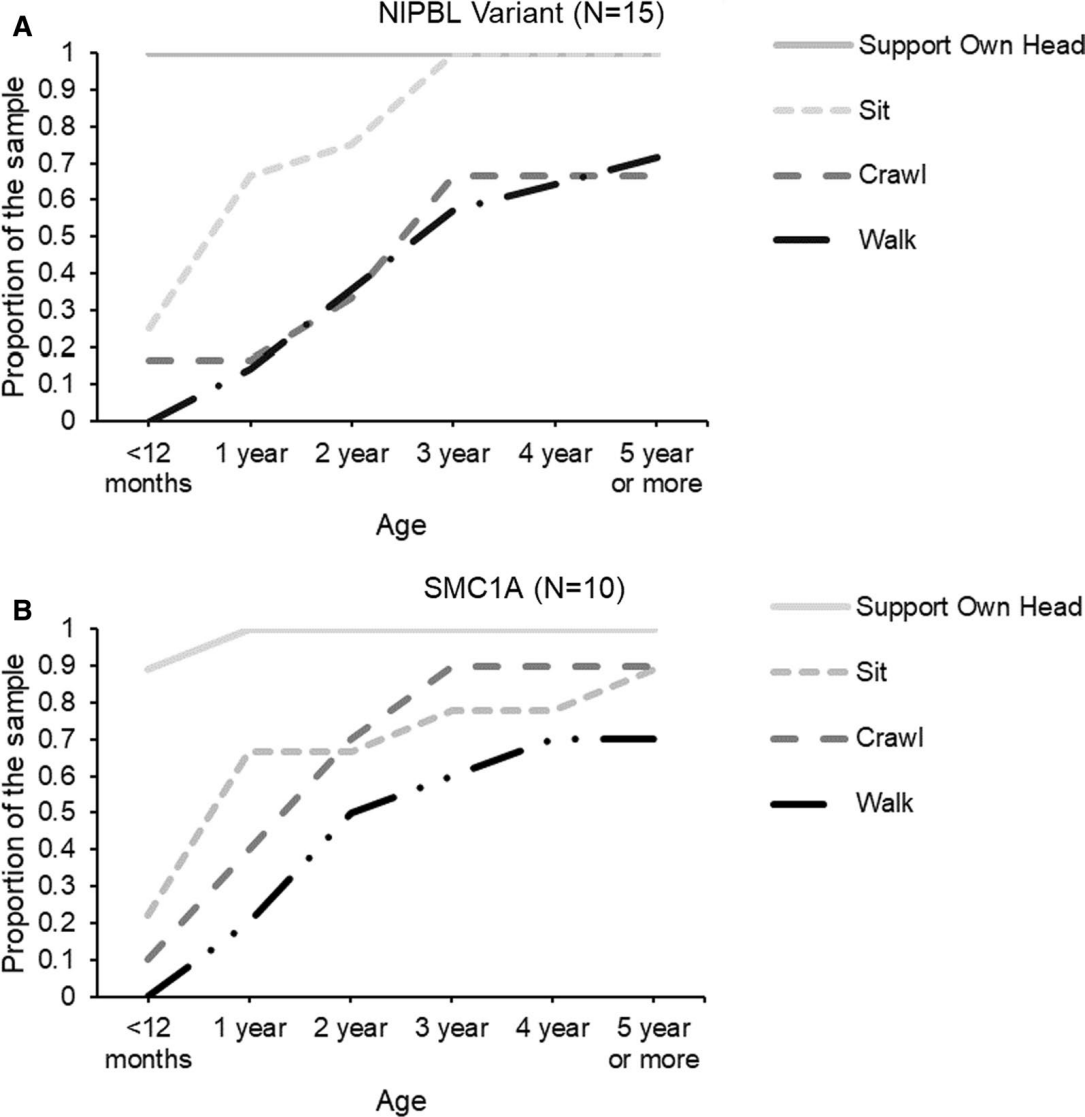
**A** and **B** Proportion of the patients who met the motor developmental milestones by 5 years of age

**Fig. 3:**
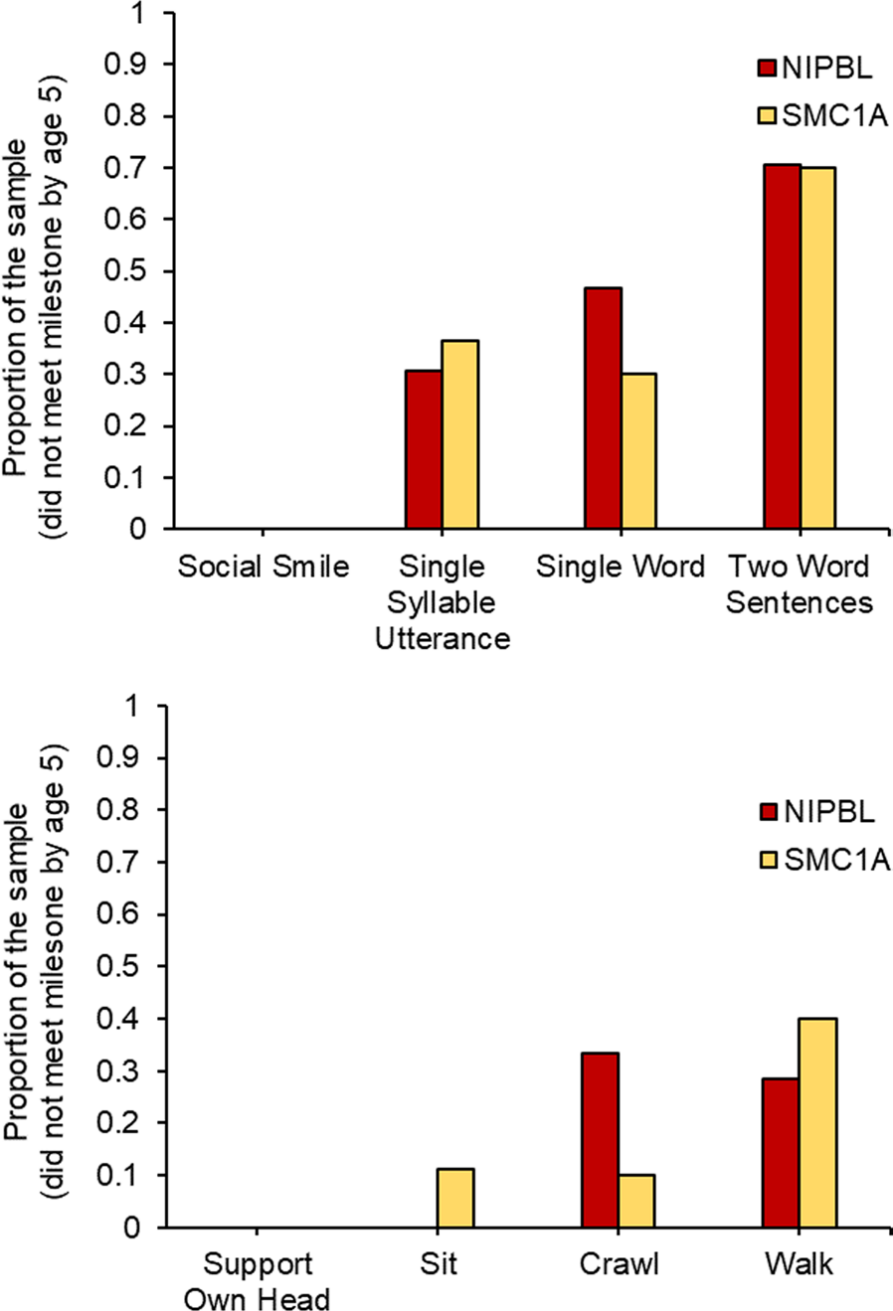
Proportion of patients with mutation in *NIPBL* or *SMC1A* who did not meet the language or motor milestone by 5 years of age

Of the whole sample, 17 caregivers of the *NIPBL* variant group and 11 of the *SMC1A* variant group completed a developmental survey regarding the timeframe their child met major motor and language milestones. Figures 1A, B and 2A, B illustrate the proportion of the clinical sample that met language and motor milestones over the first 5 years of life. No group differences were observed in the attainment of early language and motor milestones. Across the two clinical groups, the proportion of participants who did not meet later language milestones (Single Word Use, Two Word Sentence Use) were similar, with about 70% of both groups not mastering two-word sentences by the survey completion (i.e., at least 5 years of age) (Fig. 3A, B). About 30% of the *SMC1A* and 50% of the *NIPBL* variant groups were not walking unassisted by the time of survey completion, albeit the group effect did not reach significance. Notably, as outlined in Table 2, when examining the subset of participants who reported meeting the milestone, those with *NIBPL* variants were less delayed than those with *SMC1A* variant during early communication development (Social Smile, Utterance, Single Word Use). However, these patients were on average more delayed and more variable in their attainment of more complex language (Two Word Sentence Use). Both groups were similarly delayed in meeting motor milestones, albeit those with *NIPBL* variants similarly showed more variability in the age of achieving milestone for more advanced gross motor functions (Walking Unassisted). While both groups are significantly delayed relative to the developmental milestones released by the CDC [6], they show some similarities in language progression based on the published developmental data of a mixed group of individuals with CdLS [15]. However, later motor functions like Walking Unassisted yielded different developmental milestones among our groups relative to the sample in Kline et al. [15]. Among the participants with an *NIPBL* variant, Walking Unassisted was on average a year more delayed than the reported timeframe in Kline et al. [15] which may stem from different operationalization of milestones as highlighted in Discussion below.

**Table 2:** Mean age of meeting milestone (years) among the patients who reported meeting the milestone by the time of study participation. Standard deviation and range are provided in parentheses and brackets

	Center for disease control and prevention (CDC)	CdLS group [15]	NIPBL	SMC1A	Linear regression test results	
	Developmental milestones (approximate time when a child reaches a milestone)		Mean age in years (standard deviation)[range]		β, t-value, *p* value	r^2^
Communication skill development			N = 17	N = 11		
Social smile	2 Months	3 Months	0.95 (1.58)[0.5–6]	0.61(0.22)[0.5–1]	β = -0.12, t = 0.57, n.s	n.s
Single syllable utterance	–	–	1.22 (0.66)[1–3]	1.35 (0.98)[0.5–3]	β = -0.04, t = 0.15, n.s	n.s
Single word	15 Months	18 Months	1.50 (0.75)[1–3]	2.50 (1.70)[0.5–6]	β = 0.38, t = 1.43, n.s	n.s
Two word sentences	24 Months	4.5 Years	3.00 (1.87)[1–5]	3.00 (0.00)	β = -0.68, t = 6.05, *p* = 0.002	0.95
Motor development			N = 15	N = 10		
Support own head	4 Months	–	0.50 (0.00)	0.55 (0.16)[0.5–1]	β = 0.25, t = 1.18, n.s	n.s
Sit	9 Months	12 Months	1.45 (1.01)[0.5–3]	1.62 (1.57)[0.5–5]	β = 0.05, t = 0.25, n.s	n.s
Crawl	–	–	2.12 (1.09)[0.5–3]	1.72 (0.90)[0.5–3]	β = -0.20, t = 0.86, n.s	n.s
Walk unassisted	18 Months	24 Months	3.10 (2.60)[1–10]	2.14 (1.06)[1–4]	β = 0.20, t = 0.78, n.s	n.s

The table above includes milestones recently published by the CDC, which was defined as the time when 75% or more children can complete the skill by the age. Of note, these milestones are not meant to be used as developmental screening measures alone as highlighted by CDC [21], these were included to provide a qualitative comparison of our sample in addition to the heterogeneous sample of patients with CdLS published in Kline et al. [15]. The linear regression test results outlined above stem from linear regressions with age at survey completion controlled, gene variant group (*NIPBL, SMC1A*) as a predictor, and the developmental milestone as the dependent variable. The response option of < 12 months were coded as 0.5. Regression coefficient (r^2^) represents the proportion of variance of the developmental milestone explained by both the age at study participation and gene mutation

*n.s.* not significant (*p* > 0.10)

Participation in speech and occupational interventions were comparable across both groups, but those with *SMC1A* variants were more likely to engage in physical therapy (FET *p* = 0.05) and use of communication devices (FET *p* = 0.08) (Table 1).

### Behavioral phenotype

Caregivers’ ratings implicate a history of autistic features more prominently in the *NIPBL* variant group, meaning individuals are more likely affected by severe behavioral difficulties currently or in the past. Indeed, those with *NIPBL* variants yielded more elevated Repetitive Behavior Total compared to the *SMC1A* variant group but not Attention Problem Total (Table 1). Table 3 outlines the proportion of participants across variant groups that endorsed behavioral challenges. Specifically, these individuals were more likely to endorse a history of withdrawn or socially isolating behaviors (*NIPBL*: 65%, *SMC1A*: 18%), temper tantrums (*NIPBL*: 70%, *SMC1A:* 27%), hard to reach or get information through (*NIPBL*: 65%, *SMC1A*: 44%), and stereotypies like spinning (*NIPBL*: 75%, *SMC1A*: 18%). Self-injurious behaviors were observed among more than half of those with an *NIPBL* variant but none of those with an *SMC1A* variant. Likewise, those with an *NIPBL* variant were more likely to have a history of anxiety.

**Table 3:** The percentage of respondents that endorsed problem behaviors

	N (sample size)	NIPBL			N (sample size)	SMC1A			*p* Value
		Never a problem (%)	Not a problem today, but was in the past (%)	Currently a problem (%)		Never a problem (%)	Not a problem today, but was in the past (%)	Currently a problem (%)	
Presents with temper tantrums	23	30.4	0	69.6	11	72.7	0	27.3	FET *p* = 0.059
Difficult to reach or get through	20	35	25	40	9	55.6	44.4	0	FET *p* = 0.079
Isolates self	23	34.8	26.1	39.1	11	81.8	18.2	0	FET *p* = 0.015
Attention									
Excessively active	22	68.2	4.5	27.3	10	30	30	40	FET *p* = 0.063
Restless	22	45.5	4.5	50	10	30	20	50	n.s
Does not pay attention to instructions	21	14.3	9.5	76.2	10	20	20	60	n.s
Easily distractible	21	14.3	14.3	71.4	11	18.2	9.1	72.7	n.s
Repetitive/unusual behaviors									
Rocks body back and forth	24	87.5	0	12.5	11	90.9	0	9.1	n.s
Spins, twirls, paces	24	25	33.3	41.7	11	81.8	18.2	0	FET *p* = 0.004
Need to line up items or make symmetrical	24	45.8	16.7	37.5	11	81.8	0	18.2	n.s
Unable to throw things away	22	81.8	9.1	9.1	11	90.9	9.1	0	n.s

*FET* Fisher’s exact test, *n.s*. not significant (*p* > 0.10)

More respondents from the *SMC1A* group reported a history of hyperactivity (*NIPBL:* 32%*, SMC1A:* 70%). No other group differences in attention were observed although a large proportion of both samples endorsed a significant history of restlessness (*NIPBL:* 55%, *SMC1A*: 70%), challenges with paying attention to directions (*NIPBL:* 86%, *SMC1A*: 80%), and distractibility (*NIPBL:* 86%, *SMC1A:* 82%), underscoring the importance of treating attention and hyperactivity symptoms to improve quality of life.

## Discussion

To our knowledge, this is the first study focused on examining genotype–phenotype relationships in developmental and behavioral functioning among those with CdLS. Importantly, unlike many investigations involving retrospective chart reviews—which often incorporate mixed methods, clinician judgment and measurement tools—this study involved prospective standardized data collection as part of patients’ caregiver participation in a rare disease registry (CoRDS). Main findings generally suggest similar developmental delays in early communicative and motor development across those with a variant in either *NIPBL* and *SMC1A,* albeit those with *NIPBL* variants showed greater variability in the achievement of more complex language and motor milestones. Interestingly, those with *NIPBL* variants appear to present with a more severe behavioral phenotype. In brief, individuals with CdLS would benefit from early neuropsychological or neurodevelopmental assessments to identify individual developmental patterns, inform early intervention services (e.g., speech/language, physical, behavioral health therapies), and assist in connecting families to appropriate assistive technology specialists as warranted.

In contrast to prior research suggesting more cognitive delay among those with an *NIPBL* variant [7, 9, 10, 12], our findings suggest similar delays in early motor and communicative/language development. Discrepancies in findings can stem from low sample sizes and focused clinical samples (e.g., our inclusion of only individuals with gene variants and not deletions). The lack of variant status leaves challenges in our data interpretation, as we are unable to determine whether the *NIPBL* or *SMC1A* groups are largely comprised of a specific variant type. Prospective studies should consider the variant type as recent studies in CdLS show individuals with truncating variants present with more autism-related features and communication challenges than those with non-truncating (e.g., missense) variants [1]. Moreover, our study design was constrained by deidentified survey data provided through CoRDS, such that it was not possible to inquire caregivers to clarify the specific months when developmental milestones were met. Unlike prior studies on CdLS and development [15] which included more detailed review of early developmental achievements, inventories used here afford approximations of early language and motor gains but have limited sensitivity in discerning the extent of delays. For example, two children may achieve single word use at 6 months versus 11 months of age, and parents similarly check the response option of “< 12 months” when one is meeting the milestone early and the other on time. Likewise, caregivers of two children who walked at 18 months and 24 months of age may indicate they met the milestone at 2 years of age suggesting both are similarly delayed when in fact the former child achieved it broadly within normal limits. While our measures have their flaws, it is important to underscore that our study utilized a standardized approach to obtain neurodevelopmental information of patients, whereas many prior genotype–phenotype studies provide vague information as to how cognitive functioning was assessed or utilize heterogeneous measures (i.e., retrospective chart reviews with different assessments across patients). In brief, future research should consider utilizing more nuanced questions to index early developmental milestones achievements (e.g., age in months, qualitative information on developmental history), and integration of standardized developmental screening measures such as the Vineland Adaptive Behavior Scales or the Developmental Profile 4th Edition that has normative comparison data and yields age equivalents to assess the extent of delay. Indeed, recent investigations using standardized cognitive measures in older school-age children with CdLS have revealed more severe autistic features and challenges across communication and motor skills among those with *NIPBL* truncating variants than in those with missense variants [1]. Adopting a similar approach using standardized developmental assessments will be helpful to determine if such patterns are seen in early childhood or if these trends evolve over time.

It is important to note that those with *NIPBL* variants yielded greater variability in achieving more advanced language and motor milestones, if at all. This could be due to molecular factors, such as whether the variant was a missense vs. loss of function variant, or the location along the gene. However, divergent patterns between our findings with prior investigations that highlighted greater cognitive deficit in those with *NIPBL* variants [7, 9, 10, 12], may also reflect more limited developmental gains over time in these individuals compared to those with an *SMC1A* variant. In addition, the operationalization of cognitive impairment across investigations varied, as some described the extent of intellectual disability, including adaptive functioning and life skills, whereas others reported “cognitive delays” in their sample. It is also unclear if cognition was indexed by intellectual functioning, nonverbal reasoning skills, or receptive language or language comprehension particularly if a large proportion of those affected by CdLS may not master phrase or sentence speech.

To our knowledge, our study first documents differences in intervention history across genotype linked to CdLS. Those with *SMC1A* variants were more likely to use communicative devices and engage in physical therapy than those with *NIPBL* variants despite similar delays in later milestones (e.g., sentence use, walking). It is possible that more substantial early childhood delays in speech/language functions among those with *SMC1A* variants drove families to initiate developmental surveillance and treatments more promptly, thus, resulting in more timely integration of communication technology.

Additionally, more severe behavioral phenotypes, including autistic features, anxiety, and self-injurious behaviors, were observed in those with *NIPBL* variants, supporting a genotype-focused approach in treatment planning among those with CdLS. From a clinical standpoint, healthcare and school providers working with individuals with CdLS may consider integration of behavior intervention services in outpatient and school settings. Structured behavioral treatment approaches such as applied behavior analyses (ABA) may be beneficial for these children to support their social communication and behavior regulation skills. Regarding future research directions, more extensive genotype–phenotype investigations utilizing a comprehensive battery of multiple informant inventories in addition to clinical structured interviews/observations will be essential to delineate the shared versus unique behavioral traits among those with different CdLS-causative gene variants. Longitudinal investigations with larger samples of patients with CdLS-causing gene variants (e.g., inclusion of those with pathogenic variants in *HDAC8*, *SMC3,* etc.), comparison groups of idiopathic autism spectrum disorder and/or intellectual disability, and other clinical groups with overlapping CdLS-like features, such as pathogenic variants in *EP300, AFF4, NAA10,* and *TAF6*, are necessary to disentangle the different disease pathways that underline the CdLS neurobehavioral phenotype. In addition to behavioral measures, mixed methods including biological metrics such as functioning magnetic resonance imaging (fMRI), cortisol reactivity, and electroencephalogram (EEG) are vital to understand the effect of genotype anomalies on brain-behavior development. Ultimately, literature resulting from these efforts will be central to consider in later construction of clinical trials.

### Study limitations and future directions

Despite the novelty of our findings, several study limitations should be considered. Given our limited participant pool, our study had low statistical power and poor sensitivity to detect more nuanced developmental differences between variant groups. As noted above, larger samples and appropriate comparison groups are needed to clarify genotype–phenotype relationships. More comprehensive behavioral and developmental assessment measures including those with normative comparison samples, and neurobiological metrics should be applied in subsequent research in CdLS. A developmental approach should be applied in examining progress across language and motor functions, and the extent these affect higher order cognition such as abstract reasoning and problem-solving. Given our findings, detailed review of intervention history in relation to developmental surveillance should be considered to determine whether observed greater variability in later language and motor skills in those with *NIPBL* variants may stem from differences in the initiation of or intensity of treatment services. Applying a developmental framework in CdLS research would be essential to understand the effect the pathogenic variant in CdLS-causative genes has on the maturation processes of focal neural networks. So, too, comparing the specific types of variants in the various genes, juxtaposed on both development and behavior, may be of benefit.

In brief, results from this study show significant but comparable developmental delays in early childhood between those with *NIPBL* vs *SMC1A* variants; however, those with *NIPBL* variants present with more severe behavioral challenges. Findings support consideration of more genotype-focused program of treatments given differences in phenotypic severity.

## Data Availability

The data that support the findings of this study are available from CoRDS Registry but restrictions apply to the availability of these data, which were used under license for the current study, and so are not publicly available. Data are however available from the authors upon reasonable request and with permission of Sanford.
